# Building Embodied Spaces for Spatial Memory Neurorehabilitation with Virtual Reality in Normal and Pathological Aging

**DOI:** 10.3390/brainsci11081067

**Published:** 2021-08-14

**Authors:** Cosimo Tuena, Silvia Serino, Elisa Pedroli, Marco Stramba-Badiale, Giuseppe Riva, Claudia Repetto

**Affiliations:** 1 Applied Technology for Neuro-Psychology Lab, IRCCS Istituto Auxologico Italiano, 20149 Milan, Italy; e.pedroli@auxologico.it (E.P.); giuseppe.riva@unicatt.it (G.R.); 2 Department of Psychology, Università Cattolica del Sacro Cuore, 20121 Milan, Italy; silvia.serino@gmail.com (S.S.); Claudia.repetto@unicatt.it (C.R.); 3 Faculty of Psychology, Università e Campus, 22060 Novedrate, Italy; 4 Department of Geriatrics and Cardiovascular Medicine, IRCCS Istituto Auxologico Italiano, 20149 Milan, Italy; stramba_badiale@auxologico.it; 5 Humane Technology Lab, Università Cattolica del Sacro Cuore, 20121 Milan, Italy

**Keywords:** embodiment, navigation, virtual reality, dementia, mild cognitive impairment

## Abstract

Along with deficits in spatial cognition, a decline in body-related information is observed in aging and is thought to contribute to impairments in navigation, memory, and space perception. According to the embodied cognition theories, bodily and environmental information play a crucial role in defining cognitive representations. Thanks to the possibility to involve body-related information, manipulate environmental stimuli, and add multisensory cues, virtual reality is one of the best candidates for spatial memory rehabilitation in aging for its embodied potential. However, current virtual neurorehabilitation solutions for aging and neurodegenerative diseases are in their infancy. Here, we discuss three concepts that could be used to improve embodied representations of the space with virtual reality. The virtual bodily representation is the combination of idiothetic information involved during virtual navigation thanks to input/output devices; the spatial affordances are environmental or symbolic elements used by the individual to act in the virtual environment; finally, the virtual enactment effect is the enhancement on spatial memory provided by actively (cognitively and/or bodily) interacting with the virtual space and its elements. Theoretical and empirical findings will be presented to propose innovative rehabilitative solutions in aging for spatial memory and navigation.

## 1. Towards an Embodied Space Approach in Spatial Neurorehabilitation

Body-related cues (e.g., motor, vestibular, proprioceptive information; also known as idiothetic), environmental cues (e.g., landmarks, boundaries, sounds, smells; also known as allothetic), and symbolic representations (e.g., previous memories, verbal descriptions, depictions) are crucial to define cognitive maps as we actively explore the environment [1,2]. Navigational strategies (e.g., path integration, landmark-based, imagery) for determining orientation and locations rely on the successful use of at least one of these elements [3]. Indeed, spatial cognition frames of reference (i.e., body-centered representations, namely egocentric frame; world-centered representations, namely allocentric frame) that support successful spatial navigation and memory have been shown to decline in normal aging and neurodegenerative diseases [4,5,6,7,8,9]. Simultaneously with these impairments, the elderly face a decline in idiothetic and sensory processing that accounts for spatial navigation deficits in normal aging and neurodegenerative diseases [10]. Following this line of reasoning, innovative rehabilitative solutions could exploit cognitive, bodily, and environmental information to enhance spatial navigation and memory [10,11]. To pursue this aim, virtual reality (VR) is used as a powerful tool for developing personalized solutions in normal and pathological aging [12,13,14]. The sensorimotor information provided by VR navigation consists of visual information (e.g., a street with buildings) and hand motor commands (e.g., the joypad to move in the VR). Interestingly, other sensorimotor cues are being studied for navigation purposes. New solutions use the addition of the auditory and even olfactory system to gain spatial information [15,16] or exploit immersive VR systems to involve body-related information [1]. Importantly, VR provides multisensory experiences of virtual navigation [16,17] and manipulates the environmental information according to needs [15,18].

However, despite positive clinical outcomes, VR-based solutions showed some limitations, such as methodological issues and a low degree of immersion [19,20]. On the one hand, virtual symbolic cues are successfully used to explore the environment and train spatial memory; on the other hand, the studies in this field mainly used low idiothetic involvement for navigation and low use of environmental cues for orientating and remembering locations. Table 1 provides a summary of the cues involved in current VR spatial rehabilitation in aging.

Indeed, the importance of bodily and environmental information has been already highlighted by embodied cognition researchers. In contrast to the classic hypothesis, which argues that space representations are amodal [26,27], the modality-dependent hypothesis states that representations are not always abstract and can depend on the modality of acquisition [1]. It is possible to build a cognitive map of the environment (e.g., a path) from different sources (combined or alone), such as visual (e.g., landmarks, boundaries), symbolic (e.g., depictions, previous memories), and idiothetic information. This map can be retrieved through a representation that is different from the initial one and is defined as amodal since the representation of the space is independent of the type of encoding. Nevertheless, this does not exclude that body-based cues play an important role in forming and recalling maps of the space when the body is used for navigation [1,28]. Indeed, embodied cognition researchers showed that the sensorimotor system helps to encode spatial frames, influence the accuracy of spatial map formation, and prepare the body to respond to meaningful stimuli in the surroundings [29,30,31].

On the one hand, action–perception research by Gibson [32] showed that tools and environment give direct affordances (action possibilities) to the individual. On the other hand, research on embodiment (e.g., [33,34,35]) has pointed out how the body can influence cognition and behavior via sensorimotor simulation (re-enactment of previous sensorimotor and introspective states encoded during the experience with the world, body, and mind), modal priming (abstract concept activations via sensorimotor states), and direct (affective) state induction [36].

**Figure 1:**
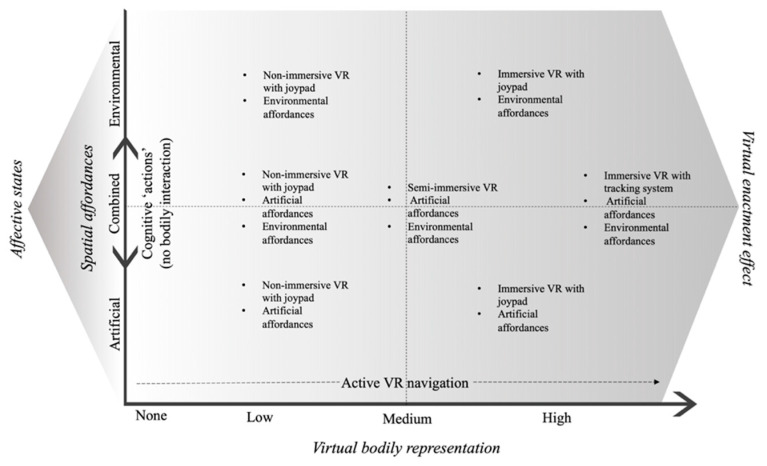
Summary of the embodied space approach and how virtual body representation, spatial affordances, and affective states could interact to foster virtual enactment effect. VR = virtual reality.

According to the notion of ‘embodied learning’ [35], learning is facilitated by using a physical propriety of the stimulus (e.g., its appearance) or by gaining knowledge around bodily activities related to the stimulus. For example, we can merely observe a stimulus (e.g., colored arrows) or we can use congruent body interaction with the stimulus (e.g., landmark) to process the navigation. Additionally, low and high levels of bodily engagement can be involved during learning activities. Therefore, we can use a congruent gesture (pointing right) when we see the stimulus (landmark or read a written instruction) as a low form of bodily engagement or conversely be involved with a whole-body interaction in the virtual space. Three elements will be presented that could be applied to VR rehabilitation for spatial memory within the framework of an embodied notion of space: virtual bodily representation, spatial affordances, and virtual enactment effect. Figure 1 provides a framework of how these elements can interact.

### 1.1. Virtual Bodily Representation

The virtual bodily representation is the combination of idiothetic cues involved during the virtual navigation, in addition to vision. These are recruited by the VR output (e.g., computer screen, visors) and input (e.g., joypad, treadmill, foot motion pad) devices to make virtual navigation more real [28,37,38]. In this sense, a navigation task where the participant actively uses cognitive functions (e.g., decides where to go, manipulates maps) but does not have control of the interface is considered as having no bodily representations in the virtual space. Conversely, a fully immersive VR represents the higher bodily representation in the simulated scenario: we can create bodily and/or cognitive representations, with different degrees of bodily cues depending on the level of virtual bodily representation in the VR (see Figure 1). These representations of the virtual world can be acted at encoding and re-enacted later for retrieval (i.e., sensorimotor simulation; see also Section 2). Indeed, a recent systematic review [39] has pointed out how using idiothetic cues during virtual navigation can lead to better spatial memory compared to ‘passive’ condition (mere observation of the path; for definition, see [2]).

In the attempt to manipulate the degree of bodily involvement and representation in the VR, Tuena and colleagues [40] showed that with VR it is possible to manipulate bodily engagement during an ‘active’ vs. ‘passive’ navigation task in a virtual city for the assessment of episodic memory. In the full-embodiment condition, the participants were immersed in the virtual city thanks to a 3D visor and navigated by walking in place (steps were detected with Kinect camera); in the medium-embodiment condition, they watched a prerecorded navigation and simulated locomotion by walking in place; and in the low-embodiment condition, they passively watched the prerecorded video with the visor. The authors found that the sense of presence (i.e., the illusion of being located in the virtual world [41]) was higher in the first condition compared to the other two, but no effect on egocentric and allocentric episodic memory arose. Similarly, Huffman and Ekstrom [26] showed that there is no difference among ‘impoverished’ (desktop and joypad), ‘limited’ (visor and joypad), and ‘enriched’ (visor and treadmill) conditions in terms of allocentric spatial orientation relative to landmarks and also in terms of medial temporal lobe involvement. They concluded that visual information might play a key role in large-scale spaces compared to bodily cues. These findings are raising an important debate [1,42] concerning how and when bodily cues influence spatial memory. Their contribution might depend on the type of the task (i.e., egocentric or allocentric), the spatial scale (i.e., peripersonal and large scale), and the type of the interface (linear vs. exponential ability to use virtual and real-world bodily cues). It might be that bodily cues are particularly relevant for egocentric and near-space representation of the environment also depending on the role of the VR interface [42] or that the ability to use cues follows an exponential trend in real-world compared to VR navigation [1].

Previous research showed that the ability to make use of bodily cues is dynamic and changes throughout the lifespan, affecting navigation and memory in aging and neurodegenerative diseases [10,11,43,44]. Interestingly, this declining idiothetic information can be used successfully to improve memory in aging. In the study of Plancher and coauthors [45], the participants had to actively navigate a virtual city through driving simulation devices compared to ‘passive’ navigation and were asked to remember details of events happening along the streets. They found that the control, mild cognitive impairment (MCI), and Alzheimer’s disease (AD) group showed improvement in allocentric memory, item memory, and binding of episodic details after the ‘active’ compared to the ‘passive’ navigation (see also Section 2). Further research is needed in this field but improving the virtual bodily representation could be a way to train a declining function in aging and use it to enhance virtual navigation tasks for spatial memory rehabilitation.

### 1.2. Spatial Affordances

Spatial affordances are cues used during navigation and in this article are classified into two groups: symbolic (e.g., maps, arrows, instructions) and environmental (i.e., discrete landmarks, boundaries; see [46]). Spatial affordances can be externally or internally generated and integrated into the navigation task. Precisely, the first type of cue is presented symbolically and can be immediately used as a compensatory aid; the second is processed by the individual according to the subjective salience of the environmental landmark. For instance, listening to instructions, reading a map, or seeing a directional arrow differ in terms of embodiment compared to the situation when we actively associate actions and decisions to a selected landmark. The first type has a low level of embodiment, whereas the latter has a high one. Interestingly, affective states can also enhance the respective level of embodiment of these cues.

For what concerns emotions, in the experiment of Ruotolo and colleagues [47], participants passively navigated a route with positive, negative, or neutral IAPS [48] photos (landmarks) at turning points. The authors showed that the participants who watched the navigation with positive landmarks were better at reordering the images and at drawing a map of the route. Conversely, the participants in the negative landmark condition rated the route as longer than the positive and neutral ones and took more time to mentally travel between landmarks. Again, in the study of Piccardi and colleagues [49], participants were required to perform a real-world navigation task (learning and recalling a path in the Walking Corsi Test) or to perform a nonembodied task (‘paper and pencil’ path drawing and landmark recognition) to study the effect of emotional (positive/negative, high arousal/low arousal) and neutral landmarks (i.e., photos from the IAPS [48]). Results showed that the embodied landmarks improved path learning, but the recall performance was the same in the two conditions.

In terms of actions paired with landmarks, Morganti [50] found that the VR version of the traditional Money Road Map test was more effective than the ‘paper and pencil’ version in providing egocentric inputs useful for orientation. In this test, the subject is asked to describe turns (i.e., left-right) by watching a path on a city-like map and cannot turn the map around to match her/his perspective with the one on the map. Participants in the VR version of the test did not have to re-locate continuously on the map; instead, landmark-based turns were done on the body axis without changing the perspective. This means that VR seems able to provide an enactive spatial representation (sensorimotor coupling with agent’s actions in correspondence of landmarks) that differs from classic neuropsychological testing. Indeed, the virtual version allows individuals to use landmarks as affordances to plan navigation. While the egocentric frame can be easily processed within an enacted and embodied approach (it is action-oriented), research also shows that independent body-based information (allocentric frame) of landmarks follows this rule [30]. In their experiment, König and colleagues [30] tested spatial allocentric maps of participants’ hometown by assessing unitary coding (angular difference between the orientation of a well-known building or street and true north) and binary coding (angular difference between the orientation of two well-known houses or two well-known streets; pointing from one well-known building to another well-known building) in spontaneous (3 s to respond) and cognitive reasoning (no time-limit) conditions. According to the authors, the former response type is thought to reflect the re-enactment of a spatial behavior (i.e., action-related information) rather than the behaviors and cognitive processes combined. Results from this study showed that binary coding is accessed intuitively when using buildings (but not streets) to generate navigation behavior and action, whereas unitary coding requires cognitive processing. They found that, when spontaneous, the retrieval of relations between landmarks (buildings) yielded better performances compared to building unitary coding. Conversely, when spontaneous action-related information is suppressed by cognitive reasoning, an inverse pattern emerged for the cardinal orientation of both buildings and streets. They concluded that allocentric information concerning landmarks’ orientation and location can be also coded within an enacted and embodied framework. Again, Cogné and colleagues [17] using a VR navigation task found that salient landmarks (e.g., elements in the environment associated with navigation decision-making and motor commands—“at the church, I have to turn right”) processing improved MCI recall compared to AD individuals. Control, MCI, and AD groups exhibited improved spatial memory when a visual navigation cue (i.e., arrow) was provided, whereas the map was helpful only in the control group. Similarly, virtual interactive maps can be used as affordances to learn spatial information of a city [51] and foster spatial frame synchronization, as shown in other studies on aging and AD [23,52,53]. In this sense, environmental cues can be conceptualized as affordances that provide external representations of the space, facilitated also by emotions, when internal ones are inefficiently computed like in aging or neurodegenerative disorders.

## 2. Virtual Enactment Effect

A way to exploit cognitive interactions in the virtual space, virtual bodily representation, and spatial affordances to improve episodic and spatial memory in aging is through the virtual enactment effect [39]. In this sense, the virtual enactment effect is a meaningful experience that arises when we actively (cognitively and/or bodily) navigate within the virtual space and with the cues proposed. This effect has a beneficial consequence because it can enhance spatial memory.

Originally, the enactment effect [54] was described as the enhancement provided by encoding sentences (e.g., open the bottle) with actual actions, instead of only reading the sentence or watching someone else performing them; the effect is thought to give deeper encoding and useful memory traces during retrieval [55]. In their review, Tuena and colleagues [39] found initial evidence of virtual enactment effect across the lifespan for spatial and episodic memory. The virtual enactment effect is the beneficial effect of actively interacting with virtual environments to provide embodied memory traces. Indeed, the review showed that sensorimotor and cognitive (i.e., active decision-making) interaction with the simulated environment had a beneficial effect on spatial memory and episodic features (e.g., ‘what’, ‘where’) in AD individuals and healthy older people. In the AD group, spared action processing allows the use of memory traces for retrieval [45]. Conversely in the latter group, the interaction with a device (e.g., joypad) overloads the executive attentional resources affecting memory recall due to dual-task processing, hence self-projected navigation through route decision-making without motor commands is preferred [44]. In young adults, the pattern is much more consistent for allocentric, egocentric, and episodic memory. The concept of virtual enactment effect could be also applied to the current debate (see, [1,42]) and be a potential explaining factor for the findings reported by Huffman and Ekstrom [26]. In their experiment, all three conditions (‘impoverished’, ‘limited’, and ‘enriched’) can be considered forms of ‘active’ navigation with different degrees of virtual bodily representation where the virtual enactment effect can occur. In this paper it is deemed crucial to compare findings against a ‘passive’ condition to answer the following questions: “Are the maps of the space truly modality-independent in ‘active’ (e.g., ‘impoverished’, ‘limited’ and ‘enriched’) and ‘passive’ conditions? Is spatial memory in these ‘active’ conditions better (i.e., virtual enactment effect) than the ‘passive’ navigation?”. Future studies could investigate which factors and mechanisms can produce the virtual enactment effect.

## 3. Conclusions

Initial studies indicate that the understanding of space processing can be improved by adopting an embodied approach. Visual and symbolic representations are surely important in building cognitive maps of the space, but body-based information should not be overlooked. However, the application of the embodied framework principles for the rehabilitation of spatial impairments is in its infancy and needs further evidence. What emerges from our overview is that, besides gold-standard techniques for memory recovery (e.g., mnemonics, vanishing cues), researchers should consider the embodied potential of bodily and environmental cues and their consequences on rehabilitation design and outcome. VR is a promising tool for the implementation of embodied pieces of training aiming at reducing the decline of bodily and cognitive information involved during ‘active’ navigation and in spatial memory in aging [2,39]. Subsequent spatial memory can be enhanced through the virtual enactment effect by improving the use of virtual bodily representation (idiothetic information), spatial affordances (environmental, symbolic cues), and cognitive activity (e.g., route-planning, spatial attention, spatial mental rotation) during navigation. Innovative sensory modalities can be added in VR: environmental auditory and olfactory cues during navigation tasks can enhance the embodied representation of the space for rehabilitation. The concepts of virtual bodily representation, spatial affordances, and virtual enactment effect can be exploited in the context of aging and neurodegenerative diseases to design spatial memory training based on enactive and grounded navigation tasks within the framework of the ‘embodied space’.

## Figures and Tables

**Table 1:** Summary of the cues involved in virtual navigation training in aging and neurodegenerative disease.

Ref.	Sample	Idiothetic Cues	Environmental Cues	Symbolic Cues	Outcome
[21]	CS	None (2D VR ‘passive’ navigation)	Virtual city with no explicit instruction to use environmental cues; ‘paper and pencil’ survey and route knowledge strategy learning	‘Paper and pencil’ maps and arrows	Results indicated that only one patient clearly improved navigation and that four correctly used the impaired navigational strategy
[22]	CS	Motor commands (2D VR with joystick)	Virtual city with no explicit instruction to use environmental cues	Map, arrows, planning list	Findings indicated that the training proposed was able to improve a wide range of cognitive functions in the virtual compared to the control group
[23]	AD	Motor commands (2D VR with joypad)	Virtual city with no explicit instruction to use environmental cues	Interactive map, directional arrows	The spatial training improved visuospatial learning test in AD
[24]	AD	Motor commands, vestibular and proprioceptive information (visor with wheelchair)	Target searching task in a virtual building	‘X’ was the target location in the building	Authors found decreased navigation errors in a single patient with AD
[25]	aMCI	Motor commands (2D VR with joystick)	Allocentric boundary-based navigation strategy; no egocentric landmark strategy	Visual feedback for correct responses	The training led to improvements in aMCI patients in episodic and spatial memory tests

AD = Alzheimer’s disease; aMCI = amnestic mild cognitive impairment; CS = chronic stroke; VR = virtual reality.
